# Research and Remedies

**Published:** 1913-08-30

**Authors:** 


### RESEARCH AND REMEDIES.

### Latent Syphilis.

With a view to getting some idea of the frequency of cases of latent syphilis—that is, where neither the history nor the symptoms and signs suggest infection—among the mothers of Melbourne, Dr. Piper arranged to have a Wassermann reaction done with the blood of one hundred consecutive cases admitted to the labour wards of the Women's Hospital there. There was absolutely no selection whatever. Sixteen positive reactions were obtained out of the hundred. Out of these sixteen patients, four were primiparæ, in whom there would otherwise have been not the very slightest suspicion of infection. Two of them were delivered of infants obviously syphilitic, but the other two babies were apparently healthy up to the time they left hospital. Happening to hold later on a post at a children's hospital, Dr. Piper chanced upon both these children attending for congenital syphilitic manifestations! He suggests that a Wassermann reaction should be done for every obstetrical case, and immediate treatment instituted whenever it is positive. He has certainly shown that latent syphilis is by no means rare, but we fear the expense of his proposal is likely to prove a bar to its adoption, to say nothing of the prejudice amongst the parents that might be encountered.

### Method of Regeneration of Corneal Tissues.

Some interesting experiments, reported in the *Archives d'Ophtalmologie*, have recently been carried out by Drs. Bonnefon and Lacoste with reference to the mode of regeneration of the corneal epithelium after injury. As the result of researches on the cornea of rabbits they find that regeneration can take place without scarring (*i.e.*, with a perfectly transparent cornea), providing that there is not a complete perforation, that the injury is done under aseptic conditions, and that the injured surface is kept free from pathogenic organisms by a proper dressing. Regeneration occurs first in the epithelium (later in the conjunctiva), there being a slight local inflammatory reaction, without apparent vascularisation, and which quickly disappears. Conjunctival regeneration is, however, constantly preceded by the appearance for a time of embryonic capillaries. Microscopic examination shows that the process of regeneration of the cornea is comparable to that which follows a loss of surface in any healthy tissue.

### The Cause of Yellow Fever.

Dr. H. Seidelin has long held that the causal organism of yellow fever is a blood parasite which has received the name of *Paraplasma flavigenum*. A year ago he had the opportunity of conducting an expedition on behalf of the Liverpool School of Tropical Medicine to Yucatan, in Central America, where an epidemic was raging. Here he carried out researches of which an exhaustive account is now published in the *Bulletin of the Yellow Fever Bureau*. He is confirmed in his belief in the specific nature of the organism, which he was enabled even to identify in his own blood during and after a quite mild attack of the fever. The exact life-history of this parasite in so far as its relation to the *Stegomyia* mosquito is concerned remains to be elucidated; but if Dr. Seidelin's observations are correct, his convictions as to the pathogenicity of the paraplasma are probably well founded. The original paper is so exhaustive that we merely note that the organism was found in "practically all" cases of yellow fever, in a number of suspicious cases, and in two apparently healthy individuals. It was not found in other febrile diseases.

### "Heretical" Views of Sunstroke.

Major W. H. Ogilvie, of the Indian Medical Service, sums up in the *Journal of the Royal Army Medical Corps* some views on sunstroke which he describes as "heretical." While admitting that he has not proved his contentions, he yet adheres very strongly to them; and there is nothing that can be said to be inherently impossible about them. He expresses his heresy thus: There is no such thing as sunstroke, other than heatstroke. Heatstroke is due, in the vast majority of cases, to non-radiation of heat from the body. This in turn is caused by deficient evaporation from the skin, and by deficient supply of cool air to the skin: of these the former is the more important. Heatstroke can also be precipitated by the heat rays of the sun adding the few fatal extra degrees to the temperature of the surface of the body. In hot and damp climates where evaporation and radiation from the skin barely suffice to keep the body temperature below the dangerous point, the heat rays of the sun are probably the deciding and precipitating factor. Incidentally, Major Ogilvie denounces in scathing terms the spinal pad for preventing sunstroke, alluding to it as a "horrible tea-cosey."

